# Scope and Spatio-Temporal Patterns of Workplace Vaccination Mandates During the COVID-19 Pandemic

**DOI:** 10.3390/ijerph23010037

**Published:** 2025-12-26

**Authors:** Claus Rinner, Mariko Uda, Laurie Manwell

**Affiliations:** 1 Department of Geography and Environmental Studies, Toronto Metropolitan University, Toronto, ON M5B 2K3, Canada; muda@torontomu.ca; 2 Faculty of Science, Wilfrid Laurier University, Waterloo, ON N2L 3C5, Canada; lmanwell@wlu.ca

**Keywords:** COVID-19, employment, government policies, occupational health, pandemic response, vaccination mandates, workplace safety

## Abstract

The global response to the COVID-19 pandemic was characterized by a patchwork of government policies in countries around the world, many of which limited civil liberties in unprecedented ways. Here, our objective was to analyze the scope and spatio-temporal patterns of workplace vaccination mandates. Using daily policy data from the Oxford COVID-19 Government Response Tracker for 2021–2022, we developed a simple mandate intensity index representing the number of affected employment sectors and the duration of each mandate by country. These metrics suggest a largely inconsistent pandemic response. We found that less than one-third of the 185 countries included in the dataset implemented such “no jab, no job” policies. Even among groups of culturally and politically aligned countries, such as the core Anglosphere, policies varied greatly: between one (United Kingdom) and 10 (Australia) out of 12 employment sectors had vaccination mandates. The most frequently and longest mandated sectors included government officials and healthcare workers, two broad groups with different risk profiles. We discuss these discrepancies from a critical perspective, considering the limited evidence for the mandates’ effectiveness along with their potential to cause harmful outcomes, and recommend careful cost–benefit analyses in the future.

## 1. Introduction

After the introduction of newly developed COVID-19 vaccines in late 2020, many countries worldwide instituted proof-of-vaccination policies and mandated vaccination of different groups aimed primarily at increasing population-level immunization rates as a pandemic exit strategy [1]. These policies were predicated on intermediate results of the manufacturers’ vaccine trials, which evaluated efficacy against COVID-19 symptoms but were not actually designed to quantify reductions in severe illness, death, or even viral transmission [2]. Nevertheless, it was generally expected that vaccine mandates would reduce disease outbreaks and that the effectiveness of mandates should be evaluated via increased vaccine uptake in target groups and the general population [3,4].

In New York City, the combination of public policies, including workplace vaccine mandates, proof-of-vaccination requirements, and financial rewards, led to an increase in COVID-19 vaccine uptake for working-age adults between July and November of 2021 [5]. It was concluded that “the increase in vaccination rates likely averted illness and death,” although no evidence to support this claim was presented ([5], p. 754). During this same period, there was a significant increase in vaccinations among racialized groups, leading to the conclusion that mandates were effective in “narrowing the gap” and reducing disparities ([6], p. 357). A reported 20% decrease in COVID-19 cases for the two months after the vaccine mandate in New York was proposed to have indirectly affected transmission rates for the highest risk groups (i.e., age 65+) in employee multigenerational households, although this was not directly assessed [7]. While some models estimated significant health and economic benefits from vaccination mandates, they also projected a very limited increase in U.S.-wide vaccination rates of only 2% by May 2022 [8].

With limited evidence of transmission reduction and growing concerns about public health ethics, COVID-19 vaccination, and specifically vaccination requirements in the workplace, became a controversial public issue. A survey by the Kaiser Family Foundation showed that a segment of the U.S. population would only accept COVID-19 vaccination if they were mandated to do so and that the general public was fairly equally divided on the issue of workplace mandates, with 51% in support and 45% opposed [9,10]. In a multi-state survey of unvaccinated long-term care network employees subjected to a vaccination mandate, 56% complied with the mandate, 21% were terminated for noncompliance with the policy, and another 21% remained unvaccinated “for other reasons” ([11], p. 1141). It was concluded that although the mandates are controversial, they significantly increased vaccine uptake, and thus “employers must weigh this benefit against the costs, including the need to terminate some employees” ([11], p. 1145).

Medical, ethical, and legal opinions also varied greatly on mandatory COVID-19 vaccinations for employment and were particularly contentious for workers in the healthcare sector. In Britain, several professional associations and councils were criticized for rejecting mandatory COVID-19 vaccinations for healthcare workers, specifically calling them coercive at a time when other European countries had already instituted them. For example, some researchers argued that healthcare workers have a specific “professional ethical duty” to contribute to pandemic “risk management” similar to other infectious disease protocols [12,13]. Similarly, Attwell et al. [14] reported general support for COVID-19 workplace vaccination mandates in the healthcare sector. Participating healthcare workers presented nuanced comments regarding medical exemptions and the need for a vaccine injury compensation program but did not support non-medical (e.g., religious) exemptions or the reassignment of unvaccinated colleagues to alternative tasks. Furthermore, adverse events arising from vaccination were characterized by one healthcare worker as an “acceptable form of collateral damage” ([14], p. 4).

While early COVID-19 vaccine mandates for healthcare, education, and federal workplaces yielded high compliance levels, they garnered stronger resistance compared to other vaccines for reasons including political polarization, immunity based on prior infection, method of enforcement, and concerns about product safety [4,15]. While many concluded that mandates were justified for schooling and employment, particularly in the healthcare sector [3,4,15], others argued that the COVID-19 vaccine mandates had far-reaching counterproductive and damaging consequences for public health, scientific integrity, trust in government, human rights protections, and socio-economic wellbeing of local and global communities [16,17]. Researchers also raised serious ethical concerns about the widespread disregard for natural immunity [18], the effectiveness of one-size-fits-all approaches [19], and general questions of public health ethics (e.g., [20,21,22]).

In 2021 and 2022, dozens of countries, including some of the most liberal democracies [17], instituted “no jab, no job” policies [16] on an unprecedented scale. According to Cameron-Blake et al. [23], Indonesia and Costa Rica had the first work mandates in February 2021, followed by Italy at the start of April 2021. By mid-June 2022, 59 countries had introduced at least one workplace mandate [23]. In this article, we aim to provide a quantitative and qualitative description of the scope and duration of workplace vaccination mandates worldwide using data from the Oxford COVID-19 Government Response Tracker (OxCGRT). We explore the geospatial patterns of workplace mandate intensity and examine its correlation with important outcomes, including COVID-19 death rates and cumulative excess mortality. Our work builds on the work by Cameron-Blake et al. [23], who examined these data but only compared the start dates of occupational mandates. Our analysis complements work on global patterns of mobility and access restrictions and other problematic implications of the authoritative COVID-19 government response policies.

## 2. Materials and Methods

We conducted an ecological, observational, cross-national time-series study using secondary policy data from the Oxford COVID-19 Government Response Tracker (OxCGRT). The OxCGRT project collected information on pandemic response measures enacted by countries around the world from 2020 to 2022, scoring the extent of each of the measures on a daily basis [24]. Their data are organized by policy indicator (25 in total) and available for 185 countries. Four policy indicators are related to COVID-19 vaccination and were first presented by Cameron-Blake et al. [23]. One of these four indicators was “V4 Mandatory Vaccination (as part of occupation or citizenship).” The V4 indicator recorded the time periods during which population groups were mandated to get a vaccine. These population groups (totalling 52) included age groups (e.g., 40- to 44-year-olds), occupation groups (e.g., staff working in an elderly care home), students at different levels of schooling (e.g., primary and secondary school students), and vulnerable groups (e.g., clinically vulnerable/chronic illness/significant underlying health condition). For each country, population group, and day, a code of 1 (requirement to be vaccinated) or 0 (no requirement to be vaccinated) was recorded.

The national vaccination policy dataset described in Cameron-Blake et al. [23] was downloaded from the final version (v1, June 2023) of the OxCGRT data repository at https://github.com/OxCGRT/covid-policy-dataset/blob/main/data/OxCGRT_vaccines_full_national_v1.csv (accessed on 7 July 2025) [24]. The spreadsheet was reduced to the V4 indicator group and the years 2021–2022. With the assumption that missing values likely signal no vaccination mandate, they were treated as zeroes. Daily scores were extracted for the 12 sub-indicators that referred to employment groups:

- V4_Airport/Border/Airline Staff
- V4_Educators
- V4_Factory workers
- V4_Frontline retail workers
- V4_Frontline/essential workers (when subcategories not specified)
- V4_Government officials
- V4_Healthcare workers/carers (excluding care home staff)
- V4_Military
- V4_Other “high contact” professions/groups (taxi drivers, security guards)
- V4_Police/first responders
- V4_Religious/Spiritual Leaders
- V4_Staff working in an elderly care home

It is important to keep in mind that the V4 data only cover government-mandated workplace vaccination, not voluntary policies enacted by individual employers or industry sectors. Where a mandate was in place but testing was offered as an alternative to vaccination, this was still recorded as mandatory vaccination by OxCGRT. As with all OxCGRT policy indicators, V4 records the presence of a policy, not its enforcement, and the indicator records the strictest policy within each national jurisdiction. For example, if vaccination was required for a particular employee group in any part of a country, this was recorded as a “1”.

The OxCGRT data table contains a column for notes, where coders could provide sources of information and explanations. There is space for only one such note per day to cover all V4 population groups. As an example, a news article titled “COVID-19 vaccine mandatory for federal workers by end of October, Trudeau announces” from 6 October 2021 [25] was referenced for Canada for 29 October 2021, the day the federal employee vaccination mandate came into effect. More details on the coding methods are available from the OxCGRT working paper [26].

The analysis comprises four main steps. We first report summary statistics such as the counts of countries with a vaccination mandate by employment sector, as well as the counts of mandated employment sectors by country. Next, for countries that enacted at least one workplace mandate at any point in 2021–2022, we created a heatmap (matrix) representing mandate duration by employment sectors and countries. We then combined the daily sector data into a simple composite index of mandate intensity per country, defined as the count of all mandate days across all sectors divided by the total number of sectors (12) and days in 2021–2022 (730):

$$\mathrm{mandate}\;\mathrm{intensity}\;\mathrm{index}=\frac{\mathrm{total}\;\mathrm{mandate}\;\mathrm{days}\;\mathrm{in}\;\mathrm{the}\;\mathrm{country}}{\left((12\;\mathrm{sectors})\times (730\;\mathrm{days})\right)}$$

We used this index to further examine the global distribution of workplace vaccination mandates and discuss individual countries with the highest index values. Lastly, we used Pearson’s correlation and simple linear regression (scipy.stats.linregress) to analyze the relationship between our index and related measures for mobility and access restrictions as well as COVID-19 outcomes. This included the OxCGRT Containment and Health Index (using the average weighted in proportion vaccinated and non-vaccinated populations [24]) as well as COVID-19 deaths and all-cause excess mortality from Our World in Data (total deaths per million and cumulative excess mortality [27]).

## 3. Results

### 3.1. Frequencies of Workplace Vaccination Mandates

Less than one-third of all countries (61 out of 185) ever implemented a COVID-19 workplace vaccination mandate in any employment sector. On average, each of the twelve sectors included in the OxCGRT dataset was mandated in 19.2 countries (median = 19). The most frequently mandated sectors were government officials (37 countries), healthcare workers/carers (35 countries), educators (27 countries), and other ‘high contact’ professions/groups (23 countries). These groups were followed by four groups mandated in 18–20 countries: frontline retail workers, unspecified frontline/essential workers, police and first responders, and elderly care home staff. Airport, border security, and airline staff, as well as military personnel, were subject to vaccination mandates in 13 and 11 countries, respectively. The least often mandated sectors were factory workers (5 countries) and religious/spiritual leaders (3 countries).

**Figure 1:**
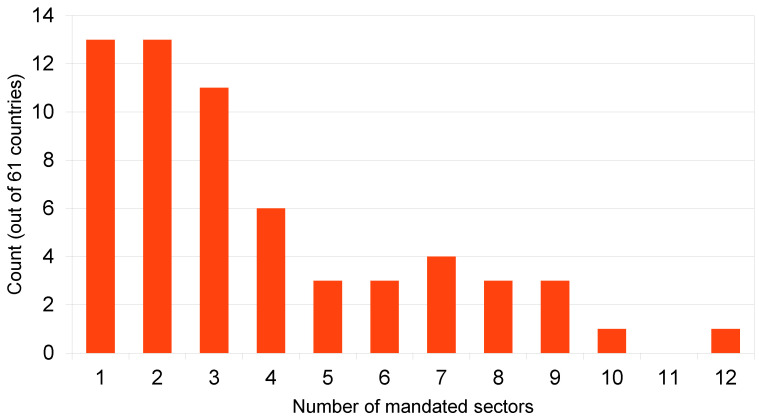
Frequency distribution of employment sector-specific COVID-19 vaccination mandates. Data source: OxCGRT [24].

Each of the 61 countries with at least one COVID-19 workplace vaccination mandate, on average, mandated 3.8 sectors (median = 3). The countries with the most sectors mandated were Tunisia (all 12 sectors); Australia (10 sectors); and Cape Verde, Kosovo, and New Zealand (9 sectors each). Another 13 countries (in groups of three or four) mandated from five to eight sectors (see Figure 1). Six countries mandated four of the 12 employment sectors. Lastly, 37 countries mandated only one, two, or three sector(s) each. If only one sector was mandated, it was government officials (4 countries), educators (3 countries), healthcare workers (2 countries), other “high-contact” professions (2 countries), Bangladesh’s mandate of religious leaders, or the United Kingdom’s mandate of elderly care home staff.

### 3.2. Duration of Mandates by Sector and Country

Workplace vaccination mandates captured in the OxCGRT data lasted anywhere between 6 days (religious/spiritual leaders in Bangladesh) and 678 days (healthcare workers in Costa Rica) during the 730-day study period of 2021–2022. The global average duration, excluding all zeroes, was 310 days or about 44 weeks (10 months). The median duration was 327.5 days, or 47 weeks, suggesting that the mandate durations are slightly skewed to the left (more low than high outliers). The average durations by sectors ranged from 209 to 361 days, with a majority falling between 267 and 305 days. The shortest average duration was found among religious/spiritual leaders (average 209 days). The longest mandated sectors included elderly care home staff (336 days), government officials (339 days), healthcare workers (353 days), and factory workers (361 days).

**Figure 2:**
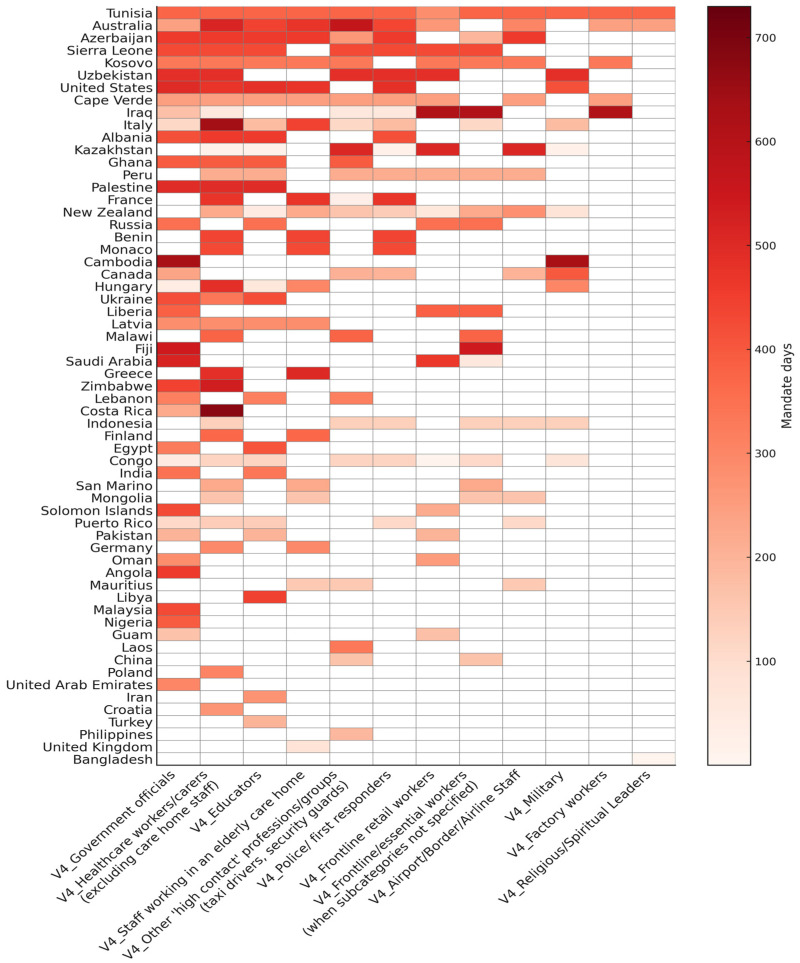
Heatmap of workplace vaccination mandates by countries and employment sectors, with colour intensity corresponding to the number of days that a sector mandate was in place in 2021–2022 (max. 730 days). Countries are sorted from top to bottom by declining mandate intensity; sectors are sorted from left to right by a corresponding metric of country counts and mandate durations. Data source: OxCGRT [24]. Countries that had no workplace mandates according to OxCGRT are not shown. Note that countries are defined by OxCGRT based on “de facto controlling authority of a jurisdiction without prejudice to conflicting authority claims” ([26], p. 71).

The heatmap (Figure 2) provides a comprehensive graphical summary of government-mandated COVID-19 vaccination in employment sectors around the globe. The countries (rows) are sorted from the top by declining mandate intensity index, and the sectors (columns) are sorted from left to right by a comparable metric that combines the count of countries mandating each sector with the duration of these mandates. Cell colours represent the number of mandate days for each country and sector. The darker the colour of a cell, the longer the mandate was in place. White cells represent the absence of a mandate.

In about half of the countries shown in the heatmap, mandates were short-lived (3–6 months) and/or limited to 2–3 employment sectors. Thus, the countries with more or extended mandates were a clear minority worldwide. Within the Anglosphere, the “Five Eyes” countries illustrate these largely divergent approaches, including Australia and New Zealand boasting 9–10 sector mandates, the United States and Canada with 5–6 sectors, and the United Kingdom with only one short mandate. The UK may thus serve as an example of a restrained approach to workplace vaccination mandates. A research brief for the British Parliament [28] explicates that as of 16 August 2021, no workplace mandates were planned other than for the care home sector, and that employers requiring staff to be vaccinated “could face a claim for unfair dismissal or indirect discrimination.” Conversely, in Canada, the governing Liberal Party was re-elected in September 2021 with a platform that included the promise of “legislation to ensure every business and organization that requires proof of vaccination from employees and customers can do so without fear of a legal challenge.” [29].

The United States and Italy, two countries with early crisis experiences in Bergamo and New York City, are further described below. Germany and Austria also had early COVID-19 hot spots in Heinsberg and Ischgl. Subsequently, Austria completely refrained from workplace vaccination mandates, while Germany required employee vaccination in two sectors: healthcare and elderly care. Germany’s “institutional vaccine mandate” (“Einrichtungsbezogene Impfpflicht”) received broad support in parliament when it was discussed and passed in December 2021, taking effect from March to December 2022. Meanwhile, Germany hotly debated and ultimately rejected a general population vaccination requirement. Austria, by contrast, is one of few countries worldwide that enacted a population vaccination mandate. However, the policy, which started in February 2022, was quickly suspended in early March 2022 before enforcement began, as the threat assessment for the Omicron variant had changed. The interactions between workplace vaccination mandates and mandates applicable to the working-age population are further discussed below.

France and Spain are two countries with global influence on the Francophone and Hispanic spheres. France mandated COVID-19 vaccination in four employment sectors, including an extended mandate from September 2021 to the end of the study period, affecting all caregiving personnel [30], including firefighters, as they may provide emergency response and transportation to vulnerable people. In March 2020, Spain had the highest excess mortality rate in Europe (54.3% [31]). Despite this early crisis situation, the country never implemented “no jab, no job” policies. Sweden, the country best known for its longer-term, strategic pandemic response, also refrained from imposing any workplace vaccination mandates.

### 3.3. Global Patterns of Workplace Vaccination Mandates

The mandate intensity index represents the proportion of sectors and time with active COVID-19 workplace vaccination mandates. For example, Tunisia’s score of 0.503 stems from the fact that the country mandated all twelve sectors for—on average—just over half of the 2021–2022 time period. This score places Tunisia at the top of the highest ten scores for mandate intensity (see Table 1).

Tunisia had a national vaccine mandate, which started on 22 December 2021, and applied to all public and private sector employees [32]. According to the OxCGRT data, the mandate was still active at the end of 2022. The coders noted that, given political unrest and lack of proper government, it is likely that the vaccine mandate was no longer enforced. However, the dataset records policy status, not implementation status.

Australia, or its states, had workplace mandates for 10 of the 12 employment groups and lengthy mandates for several groups, which led to its high score of 0.427. Mandates were most intense in the state of Victoria, where, starting on 22 October 2021, most workers were required to be vaccinated, including religious workers [33]. Australia curiously had the longest mandate in the world for the group “other high contact professions/groups (taxi drivers, security guards).” This was in part due to quarantine workers, mandated early on in May 2021 in Western Australia, being placed in this category.

The next group of countries, Azerbaijan, Sierra Leone, Kosovo, and Uzbekistan, had 6–9 mandated employment sectors each and national mandates that started in September/October 2021 (or February 2022 for Kosovo, according to OxCGRT, although no reference for that date was provided). These mandates were still in place at the end of 2022. Note that Azerbaijan appears to have only required at least 80% of employees, rather than all employees, within an organization to be vaccinated and allowed certificates of immunity against COVID-19 as an alternative to vaccination [34].

In the USA, the country with the 7th highest score, mandates and their timing varied significantly by state. The earliest mandates started in August 2021 (state employees in certain states), and most mandates lasted until at least the end of 2022. The only group to have its mandate removed slightly earlier was the military [35]. The following country in the top-10 list, Cape Verde, had a mandate applicable to a broad range of workers (9 of the 12 sectors) starting on 24 August 2021 [36] and lifted on 27 April 2022. Meanwhile, Iraq had very long mandates for factory workers, retail workers, and workers in some other businesses starting from 1 May 2021, which contributed to its high score. Note that there were some inconsistencies in the Iraq data for other groups (e.g., government officials, healthcare workers, police). Completing the most-mandated countries, Italy has a high index score in part because it had a very long mandate for healthcare workers, having been the first country in Europe to mandate this sector on 1 April 2021 [37]. This mandate was still in place at the end of 2022. For some of the time, Italy also implemented a broad mandate that applied to all public and private workers [38], which is under-reported in the OxCGRT data in the authors’ opinion.

**Figure 3:**
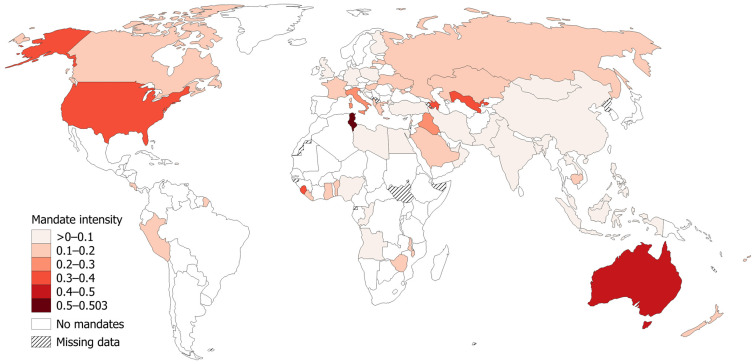
Geographic distribution of COVID-19 workplace vaccination mandate intensity (calculated as the relative number and duration of employment sector mandates in 2021–2022). Data source for index calculation: OxCGRT [24]. Note that countries are defined by OxCGRT based on “de facto controlling authority of a jurisdiction without prejudice to conflicting authority claims” ([26], p. 71). Geographic data source: Natural Earth, free vector and raster map data @ naturalearthdata.com.

Figure 3 illustrates the geographic distribution of existence and duration of COVID-19 workplace vaccination mandates for countries around the globe using the mandate intensity index. Due to the presence of workplace mandates in only 61 countries, spatial patterns are difficult to distinguish visually. Canada and the USA, occupying most of North America’s landmass, make this area appear as a cluster. Much of Asia, again visually dominated by a few large countries such as Russia, China, and India, also appears as a hot spot for workplace mandates. Additional small clusters of mandate-intense countries exist in Southern Europe, the Middle East, Africa around the Gulf of Guinea, and Oceania.

### 3.4. Correlation of Workplace Mandates with Related Public Health Measures and Outcomes

**Figure 4:**
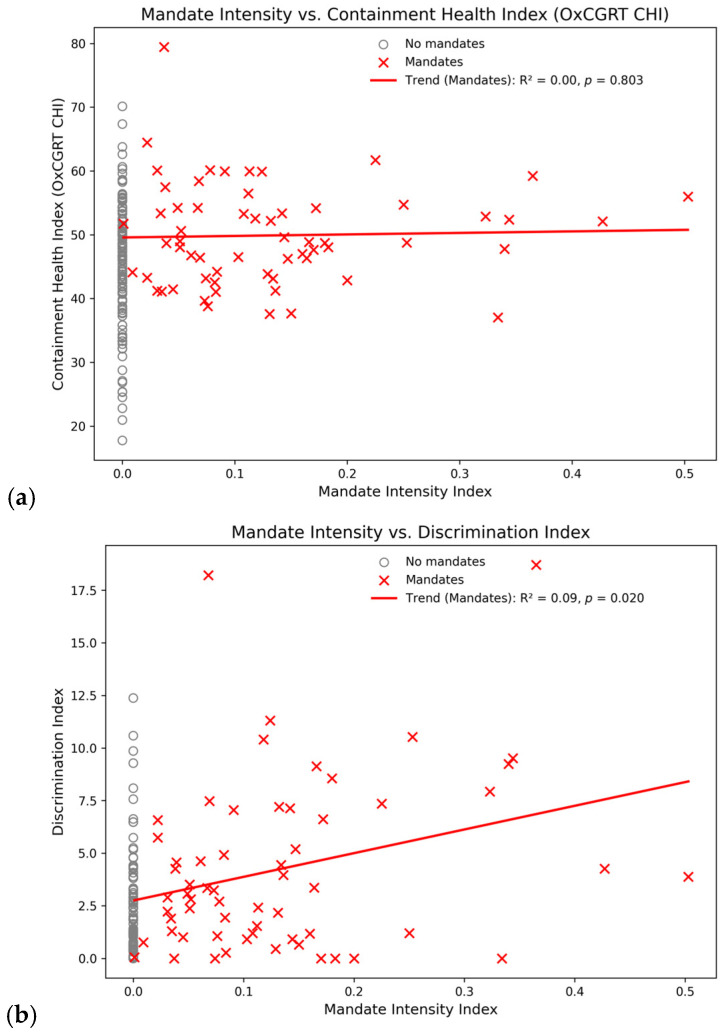

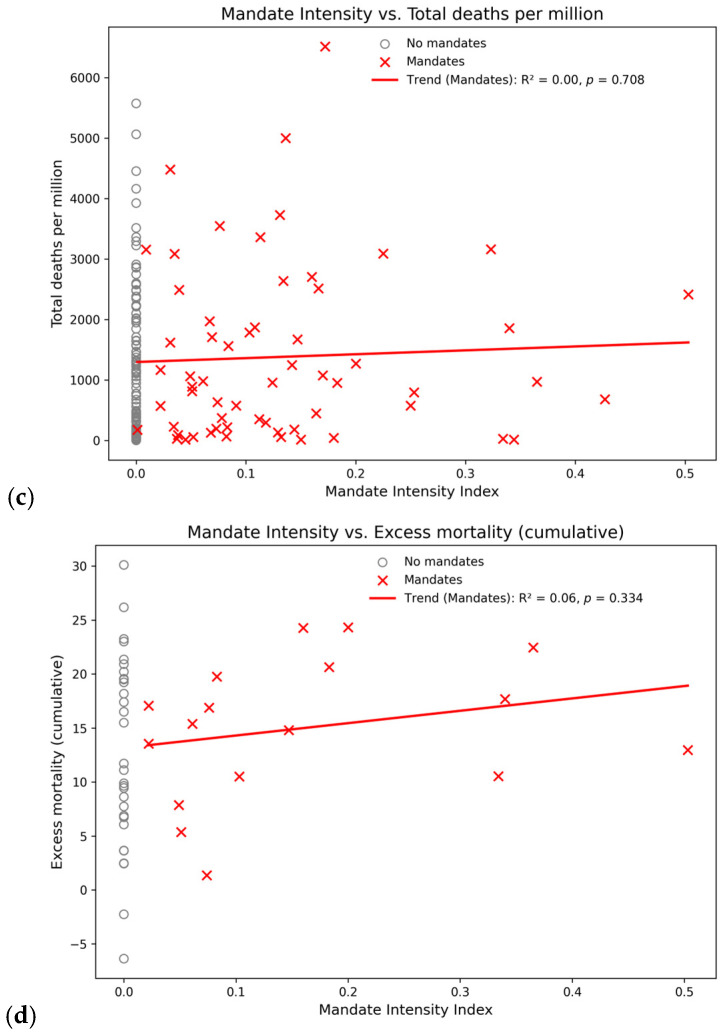
Association of workplace mandate intensity with related public health measures and outcomes: (**a**) OxCGRT Containment and Health Index [24]; (**b**) Discrimination Index [39]; (**c**) OWID total COVID-19 deaths per million [27]; (**d**) OWID cumulative excess mortality [27]. The 61 countries with at least one workplace mandate are shown as red crosses; countries without mandate are shown in grey. Linear trendlines are shown only for countries with a mandate. Note that excess mortality data were available for only 47 out of 185 countries.

Hale et al. [26] presented the Containment and Health Index (CHI) as a measure of the level of mobility and access restrictions applied by the government to all citizens. For the 2021–2022 study period, our workplace mandate intensity index correlates positively with the CHI, albeit with an extremely weak R-square value of only 0.03 (*p* = 0.030). When countries without workplace mandates are excluded, the R-square diminishes to 0.0, and the relationship is no longer significant (*p* = 0.803). The numeric overview in Table 2 and the corresponding scatterplot in Figure 4a illustrate the lack of a meaningful relationship, which suggests that workplace vaccination and broader mobility and access restrictions for all citizens were used independently of each other.

Workplace mandate intensity also correlated positively (R^2^ = 0.15, *p* < 0.001) with Rinner et al.’s [39] discrimination index, a measure based on the difference in mobility and access restrictions applied to unvaccinated vs. vaccinated individuals. When limited to the 61 countries with workplace mandates, the relationship further weakens but remains significant (R^2^ = 0.09, *p* = 0.020). The point distribution in the scatterplot in Figure 4b suggests that some governments and public health authorities implemented complementary policies marginalizing individuals who did not receive a COVID-19 vaccine.

The correlations of workplace vaccination mandates with two key public health outcomes were positive but not statistically significant; see Table 2. In these cases, the upward slopes of the trendlines in Figure 4c,d are “unexpected” (see also [40]), that is, a public health measure (workplace vaccination mandates) is associated with the opposite outcome of what it was intended to achieve. For example, countries with more intense workplace mandates tended to have higher excess mortality, and this relationship became stronger (albeit on a very low level) when focusing on the 61 countries with non-zero mandate intensity. The high *p*-values (>0.05), however, indicate that workplace vaccination mandates did not have demonstrable negative (nor positive) impacts on total recorded COVID-19 deaths by 31 December 2022 nor on cumulative excess mortality by the same date.

## 4. Discussion

As the authors are based in a highly mandated jurisdiction, it was surprising to find that only a third of countries (61 out of 185) in the world enacted COVID-19 workplace vaccination mandates and that many of these mandates were short-lived and/or limited to 2–3 employment sectors. Only some 30 countries implemented a greater number of workplace mandates or maintained them for extended periods. The countries with the highest mandate intensity scores did not cluster in any particular region but were distributed seemingly randomly throughout the globe. The four most frequently mandated sectors globally were government officials, healthcare workers, educators, and other ‘high contact’ professions. Meanwhile, the four employment sectors that had the longest average mandate durations were factory workers, healthcare workers, government officials, and elderly care home staff. Thus, when it comes to occupational vaccination mandates, the global COVID-19 response resembles a patchwork of different policies enacted at different times for different durations.

This lack of global agreement or coordination may be explained by the scant scientific evidence supporting workplace vaccination. Possible goals of mandates include protecting one or more of the following groups: individual employees; other employees and the employer from secondary effects; and customers and patients interacting with employees [41]. A widely publicized focus of the COVID-19 pandemic response was to reduce the exposure of vulnerable individuals as workers or service users at high-contact locations such as hospitals, doctors’ offices, elderly care homes, or essential retail. This concern is reflected in three of the most frequently and longest mandated workplaces (healthcare, elderly care, and other high-contact professions). In some countries, factory workers may have been subject to vaccination mandates due to crowded working conditions. Lastly, government and education workers appear to have been required to be vaccinated primarily in an attempt to increase vaccine uptake in their local communities or in response to concerns raised by vocal subsets of employees. For example, the vast majority of individuals on post-secondary campuses—young adults in their twenties—were at low risk of illness but disproportionate risk of vaccine adverse events [42,43], yet pressure from public health authorities and vulnerable faculty members led to extended vaccination mandates on college and university campuses.

Unfortunately, these justifications for mandates rely on the effectiveness of the available vaccines. In terms of COVID-19 vaccine efficacy, it was known in 2021 that viral transmission rates post-infection were similar between vaccinated and unvaccinated individuals [44], thus making vaccination mandates “inherently punitive, discriminatory, and coercive” as a condition of employment and access to healthcare and travel ([17], p. 2). In addition, it was shown that infection-derived immunity was as effective as vaccination for preventing severe disease; thus, the failure to recognize and accommodate employees with such naturally acquired immunity led to reactance effects, including distrust in the “entire public health establishment” ([17], p. 3).

In fact, political rhetoric that moralized vaccination and scapegoated unvaccinated people for the pandemic fuelled stigma and discrimination, particularly in the workplace, where mandates were used primarily as a public health strategy to increase overall vaccination rates [17]. Such politicization likely influenced increased refusals of employers to consider religious, medical or philosophical exemptions or provide reasonable accommodations such as rapid testing or work reassignment, further excluding people from the human right to work and earn a living wage [17,45]. In a series of articles, Chaufan and colleagues documented the detrimental effect of COVID-19 vaccination mandates in the healthcare sector on patient care [46,47] and the lived experiences of healthcare workers in Ontario, Canada [48]. The severe impacts for non-compliant individuals in Canada often included the loss of unemployment benefits in addition to job loss. Similarly, Monaghan & Begley [49] interrogate the life-changing consequences for unvaccinated health sciences students in Ireland, who were excluded from clinical placements, a key component of their degree requirements. The divisiveness of pandemic response policies reached a climax with the Canadian “Freedom Convoy”, a multi-week rally in the national capital, Ottawa, triggered by vaccination mandates in the cross-border transportation sector (e.g., [50]).

A brief review of the legal and scientific foundations for vaccination mandates in the USA argued that a small but significant proportion of citizens declining COVID-19 vaccinations could be addressed through education via trusted public officials and employer mandates that complied with anti-discrimination laws (e.g., for reasons of disability, medical condition, or religion) [51]. Furthermore, advisement from the US Centers for Disease Control and Prevention (CDC) and Food and Drug Administration (FDA) to federal officials was that because the COVID-19 vaccines were experimental and only permitted under the Emergency Use Authorization, they could not be legally mandated, and that, rather than enacting coercive workplace mandates, public health strategies for the pandemic should be “predicated on prevention and persuasion grounded in science before resorting to compulsion” ([51], p. 1064). In terms of religious accommodations to any vaccine mandates, they should be a “good-faith interactive process” between the employee and employer ([52], p. 9).

Considering human rights, the legality of work-related mandates, and whether such measures would be acceptable to their citizens, many governments refrained from enacting COVID-19 workplace vaccination mandates. Switzerland, a democratic country that cherishes freedom of choice, did not mandate the vaccines, and healthcare practitioners there were, in general, opposed to vaccination mandates [53]. In Mexico, employers were not permitted to mandate the vaccines [54], and the president was vocal about vaccination being voluntary [55,56].

Another factor governments possibly considered was the likelihood of voluntary vaccine uptake by citizens without mandates. Japan did not mandate due to its constitution, its government website stating, “Even if your company asks you to get vaccinated, you can choose not to if you do not want to,” but achieved high vaccination rates nonetheless partly due to peer pressure, demonstrating that mandates were not necessarily needed [57].

Governments additionally had to consider the possible loss of labour should workers leave due to mandates. In the UK, the mandate for healthcare workers was revoked on 15 March 2022, just before it was to come into effect, due to warnings of critical labour shortages if the plan went ahead [58].

Finally, work-related mandates were probably never considered in countries with leaders who themselves were vaccine-hesitant and where vaccine uptake was slow. For example, the Eritrean government did not provide any COVID-19 vaccines to its citizens from 2021 to 2023 [59]. The Burundi government was also not highly supportive of the vaccines and did not introduce vaccination until October 2021, cautioning citizens that they would take the vaccine “at their own risk” [60].

Our analysis is subject to a number of limitations. Firstly, workplace mandates may be impacted by the presence or absence of general population mandates. The OxCGRT data suggest that eleven countries required vaccination of parts or all of their working-age population. Most significantly, while there were no workplace mandates in Ecuador, South Sudan, Tajikistan, and Turkmenistan, general population mandates applied to the working-age population in these countries for extended periods of 1 to 1.5 years. In addition, Indonesia, which had six short workplace mandates, also had a general population mandate in place for ~1.5 years. For these five countries, the zero or low workplace mandate intensity scores in our analysis should be interpreted with caution.

In interpreting the OxCGRT data, it is important to understand that the data recorded policy, not enforcement. In addition, it recorded government policy, not voluntary actions taken by employers to mandate their employees. Thus, our results may not reflect the full extent of the vaccination pressure that workers experienced. For example, in the province of Ontario, Canada, post-secondary institutions were required to implement a COVID-19 vaccination policy that included antigen testing as an approach to allow students and employees to remain unvaccinated. Yet, all institutions decided to require vaccination for campus access, since the testing alternative was marked as optional in the provincial government’s policy template [61]. Thus, Ontario’s Chief Medical Officer of Health could later claim that he did not require vaccination in the post-secondary sector, yet a de facto mandate was in place from September 2021 to February 2022 or longer, based on the over-compliance of the sector’s institutions.

In addition, OxCGRT coding rules [26] stipulated that a vaccination mandate was to be recorded even if the respective policy allowed regular testing as an alternative. Some countries allowed testing in some sectors while others did not, and this is not differentiated in the OxCGRT data. Moreover, the OxCGRT data do not distinguish how many vaccine doses were required to be deemed “vaccinated.” Thus, our results just reflect the presence of a mandate and its length, and not its particulars, which can be significant.

Another issue to be aware of with the OxCGRT data is that the one employment group “frontline/essential workers (when subcategories not specified)” can act as a proxy for a number of other work categories combined. Thus, not all OxCGRT employment groups are mutually exclusive. This could lead to an underestimation of mandate intensity for countries that had mandates for this category.

According to Cameron-Blake et al. [23], some variance and error are expected in the OxCGRT vaccine indicator data due to heterogeneous interpretation of source data, lack of public information, and language barriers. Through quality control exercises, they “determined that 90% of entries were consistently interpreted” ([23], p. 1410) and “90% of the original data entries in the database were correct” ([23], p. 1411). We generally agree with these estimates. In a review of the data for 13 countries, we found a number of omissions. For example, France had a military mandate in 2021, Germany also had a military mandate, and Israel had a Green Pass, which acted like a de facto workplace mandate for a variety of employee groups (with proof of prior infection or testing allowed). However, we also discovered from looking at subnational datasets available for six countries in addition to the national datasets used in this study that mandates were missing for Brazil, Canada, China, India, and the USA, according to the OxCGRT coding rules. The OxCGRT team used a “strictest-in-the-land” rule, where for a given employment sector in a given country on a given day, a mandate anywhere within the country was supposed to be recorded at the national level, but this was not always the case. Importantly, all of these OxCGRT data errors resulted in the underestimation of mandate intensity for the corresponding countries.

## 5. Conclusions

Based on daily records of occupational vaccination mandates for 12 groups of employees from the Oxford COVID-19 Government Response Tracker, we found that less than one-third of the 185 countries included in the dataset implemented any “no jab, no job” policy in 2021 or 2022. The counts of countries with a vaccination mandate by employment sector and of mandated employment sectors by country, as well as the mandate durations, further illustrated large inconsistencies in this element of the global pandemic response. The global patterns of our composite index of mandate intensity by country were similarly unpredictable. Correlations between the index and related measures of mobility and access restrictions, as well as COVID-19 deaths and excess mortality, were not statistically significant.

We discuss these findings from a critical perspective, considering the limited evidence for the mandates’ effectiveness along with their potential to cause harmful outcomes. Moreover, significant errors in the OxCGRT dataset underestimate the burden of workplace mandates. Further research could examine select jurisdictions in more detail, with special attention to subnational government policies. The interactions between government policies and private sector decision-making on employee vaccination also merit closer attention. Overall, this research adds yet another question mark to the prevailing narrative of a successful COVID-19 pandemic response.

## Figures and Tables

**Table 1:** Country-level jurisdictions with the highest mandate intensity scores. Data source for index calculation: OxCGRT [24]. * Note that countries are defined by OxCGRT based on “de facto controlling authority of a jurisdiction without prejudice to conflicting authority claims” ([26], p. 71).

Mandate Intensity Index	Country *	Rank
0.503	Tunisia	1.
0.427	Australia	2.
0.365	Azerbaijan	3.
0.344	Sierra Leone	4.
0.340	Kosovo	5.
0.334	Uzbekistan	6.
0.323	United States	7.
0.253	Cape Verde	8.
0.250	Iraq	9.
0.225	Italy	10.

**Table 2:** Linear regression between workplace mandate intensity and related public health measures and outcomes. Calculations by the authors. Data sources for benchmarks: [24,27,39].

	Non-Zero Countries		All Countries	
Workplace Mandate Intensity vs. Related Indicators	*p*_Value	R_Squared	*p*_Value	R_Squared
Containment and Health Index	0.803	0.00	0.030	0.03
Discrimination Index	0.020	0.09	<0.001	0.15
Total COVID-19 deaths per million	0.708	0.00	0.280	0.01
Excess mortality cumulative	0.334	0.06	0.224	0.03

## Data Availability

The original data are available from the sources cited in Section 2.
